# Neoantigens Derived from Recurrently Mutated Genes as Potential Immunotherapy Targets for Gastric Cancer

**DOI:** 10.1155/2019/8103142

**Published:** 2019-06-13

**Authors:** Jie Zhou, Wenyi Zhao, Jingcheng Wu, Jun Lu, Yongfeng Ding, Shanshan Wu, Haiyong Wang, Ding Ding, Fan Mo, Zhan Zhou, Lisong Teng, Shuqing Chen

**Affiliations:** ^1^Zhejiang Provincial Key Laboratory of Anti-Cancer Drug Research, College of Pharmaceutical Sciences, Zhejiang University, 866 Yuhangtang Road, Hangzhou, 310058, China; ^2^Institute of Innovative Medicine, College of Pharmaceutical Sciences, Zhejiang University, Hangzhou 310058, China; ^3^Department of Surgical Oncology, The First Affiliated Hospital, School of Medicine, Zhejiang University, 79 Qingchun Road, Hangzhou, 310003, China; ^4^Hangzhou Neoantigen Bio-Tech Ltd., Co., 688 Road Binan, Hangzhou, 310051, China; ^5^Formulation & Analysis Lab, Hisun Pharma (Hangzhou) Co. Ltd., Xialian Village, Xukou Town, Fuyang, Hangzhou, 311404, China; ^6^Vancouver Prostate Centre, University of British Columbia, 2660 Oak Street, Vancouver, BC, Canada V6H 3Z6

## Abstract

Neoantigens are optimal tumor-specific targets for T-cell based immunotherapy, especially for patients with “undruggable” mutated driver genes. T-cell immunotherapy can be a “universal” treatment for HLA genotype patients sharing same oncogenic mutations. To identify potential neoantigens for therapy in gastric cancer, 32 gastric cancer patients were enrolled in our study. Whole exome sequencing data from these patients was processed by TSNAD software to detect cancer somatic mutations and predict neoantigens. The somatic mutations between different patients suggested a high interpatient heterogeneity. C>A and C>T substitutions are common, suggesting an active nucleotide excision repair. The number of predicted neoantigens was significantly higher in patients at stage T1a compared to in patients at T2 or T4b. Six genes (*PIK3CA*,* FAT4*,* BRCA2*,* GNAQ*,* LRP1B*, and* PREX2*) were found as recurrently mutated driver genes in our study. Combining with highly frequent HLA alleles, several neoantigens derived from six recurrently mutated genes were considered as potential targets for further immunotherapy.

## 1. Introduction

Since the approval of trastuzumab as a treatment for* HER2*-positive breast cancer in 1998 by the FDA [1], tumor-associated antigens such as CD molecules,* VEGF*, and* EGFR* have been actively targeted for drug development by the pharmaceutical industry [2–4]. The side effects of therapies based on monoclonal antibodies are mild and tolerable. However, when coupled with antibody-drug conjugates (ADC) or the chimeric antigen receptor T-cells (CAR-T) technology, the nonspecific and durable off-target cytotoxicity can be fatal for patients [5]. Therefore, the development of an optimal tumor-specific target that could differentiate tumor cells from normal tissues is essential.

Several studies have shown that targeting neoantigens in T-cell-based immunotherapy is a promising approach for treatment of lung adenocarcinomas [6], leukemia [7], and melanoma [8, 9]. Cancer is initialized by somatic driver mutations and other genetic instabilities, which are the molecular basis of the carcinogenesis process. In particular, point mutations are directly involved in essential cellular activities and functions, such as proliferation, apoptosis, and tumorigenesis. Mutant proteins are also processed by the intracellular repair system through ubiquitination and hydrolysis in the proteasome. Hydrolyzed peptides (length of 8-11 amino acids) are bonded with class I major histocompatibility complex (MHC) molecules and are presented on the cell surface as tumor-specific neoantigens, which are recognized by T-cells, provoking an immune response.

Gastric cancer (GC) is the third leading cause of cancer mortality in world. It is a common cancer prevalent in Eastern Asia, Central and Eastern Europe, and South America. The prognosis remains poor with a 5-year overall survival rate at 30.4% [10, 11]. Besides traditional chemotherapy agents, only trastuzumab, ramucirumab, and apatinib have been approved for advanced or metastatic GC. Systematic molecular profiling of GC on 595 patients by the Cancer Genome Atlas (TCGA) [12] and Asian Cancer Research group (ACRG) [13] shows that CG are highly heterogenous, exhibiting high chromosomal instability, hypermethylation, and mutation burden. Based on its molecular characteristics, the identification of neoantigens against recurrently mutated oncogenes is feasible, using current next-generation sequencing (NGS) platforms and bioinformatic analysis pipeline.

Previous studies have used genomic data from the TCGA, Foundation Medicine Adult Cancer Clinical Dataset (FM-AD), and their own cohorts to characterize neoantigens and their association with genetic alteration or with survival [14–17]. However, these studies did not focus on neoantigen profiling for gastric cancer patients. We analyzed the characteristics of somatic mutations and neoantigens, especially their correlation with clinical features of patients. The important neoantigens and their associated oncogenes shared by several patients were chosen with the goal of further developing T-cell-based immunotherapy such as vaccines for patients. The work presented here collected tumor tissues and peripheral blood samples from 32 gastric cancer patients. The whole exome sequencing was performed on Illumina Hiseq4000 sequencing system. An in-house developed integrated software “Tumor-Specific Neo-Antigen Detector” (TSNAD) [18] was used to predict neoantigens.

## 2. Materials and Methods

### 2.1. Patients

Fresh or FFPE-embedded primary tumor tissues and paired peripheral blood were collected from 32 gastric cancer patients during the period from August 12, 2016, to March 14, 2017. Among the 32 gastric patients, 11 were female patients and 4 were below 45 years of age. Of these, 2 were T1a, 6 were T2, 6 were T4a, and 18 were T4b cases, respectively. Detailed information of these samples is listed in Table 1. The enrolment of human subjects in this study was done after informed consent forms were signed. Written consent for the collection and use of tissues for research purposes has been obtained, with ethical approval from Research Ethics Committee of the First Affiliated Hospital, Zhejiang University School of Medicine, China. All methods reported in our study were performed in accordance with the relevant guidelines and regulations.

### 2.2. Whole Exome Sequencing

DNA was extracted from the tumor tissues and peripheral blood using AxyPrep Blood Genomic DNA Kit and AxyPrep Multisource Genome DNA Kit. Exomes were captured from 750 ng of genomic DNA per sample using the Agilent SureSelect Human All Exon V5 Kit (Agilent Technologies) according to the manufacturer's instructions. Paired-end multiplex sequencing was then performed on the Illumina HiSeq4000 sequencing platform. On average, the sequencing depth was 86× per sample with standard deviation ± 46×.

### 2.3. Pipeline for Somatic Mutation Analysis, HLA Genotyping, and Neoantigen Prediction

The raw data was processed by integrated software TSNAD (available on http://github.com/jiujiezz/TSNAD) [18]. This software was developed by our laboratory with a graphical user interface, which combines the necessary algorithms to identify cancer somatic mutations, determine HLA genotyping, and predict neoantigens. TSNAD can identify cancer somatic mutations following the best practices of the genome analysis toolkit (GATK) from the genome/exome sequencing data of tumor-normal pairs and also determine HLA genotyping by SOAP-HLA [19]. Then, TSNAD invokes NetMHCpan [20] to predict neoantigens which can bind to class I MHC molecules. Besides, TSNAD can also identify germline mutations.

### 2.4. Statistical Analysis

The statistical analyses were performed using R software and Wilcoxon rank-sum test was used to determine the significance. The significance was defined when p<0.05.

## 3. Results and Discussion

### 3.1. Intrapatient Heterogeneity of Somatic Mutations and Neoantigen Number

**Figure 1:**
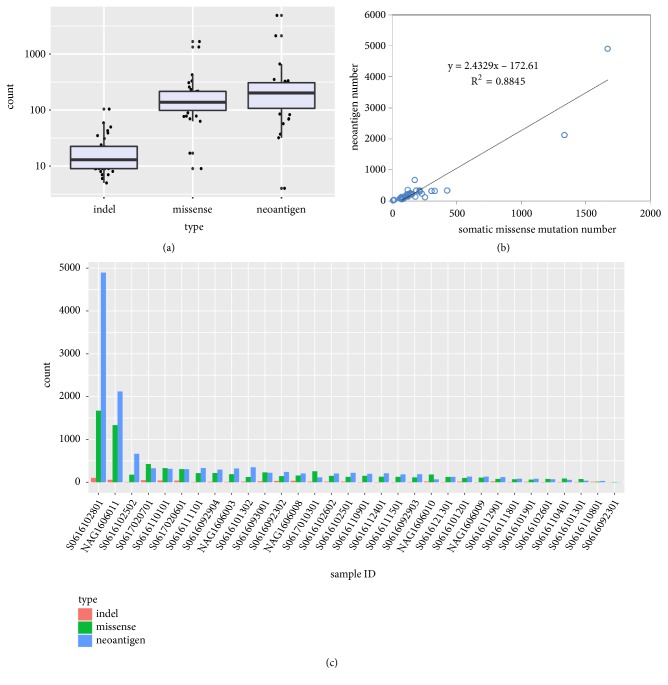
The overall number of somatic mutations and neoantigens. (a) The boxplot shows the numbers of indels, missense mutations, and predicted neoantigens. (b) The fitted curve between the number of somatic mutations and neoantigens, R^2^=0.8845. (c) The histogram of numbers of indels, missense mutations, and neoantigens for each patient.

The somatic mutation analysis and neoantigen prediction of 32 gastric cancer patients were directly performed by TSNAD from whole exome sequencing raw data. The numbers of nonsynonymous mutations, indels, and neoantigens were listed in Table 1. In total 7,432 somatic missense mutations, 658 indels, and 12,929 neoantigens were filtered by the software for 32 patients. The median number is 138 for missense mutation (median tumor mutation burden, median TMB = 4.6 mutations/Mb), 14 for indels, and 202 for neoantigens (see Figures 1(a) and 1(c)). The fitting curve revealed a laniary positive correlation between missense number and predicted neoantigens (R^2^=0.8845, see Figure 1(b)). According to three gastric cancer projects in ICGC Project (GACA-CN, GACA-JP, and STAD-US), the TMB of Chinese gastric cancer patients (median TMB = 8.467 mutations/Mb) is greater than the Japanese (median TMB = 6.467 mutations/Mb) and American (median TMB = 5.3 mutations/Mb). The difference in the tumor mutation burden between our cohort and GACA-CN is caused by the high heterogeneity of gastric cancer and the limited small sample size of our cohort.

However, there is a large variation in the number of somatic mutations between the patients studied, indicating significant molecular heterogeneity within gastric cancer. 71.9% (23/32) of patients had less than 200 missense mutations in coding regions, but there were two female patients (6.25%) who surprisingly had more than 1300 mutations (high tumor mutation burden, TMB > 40 mutations/Mb) without any experimental bias. The difference of predicted neoantigen number is similar to mutation number. The number of indels varies from 5 to 104. The majority of patients had less than 300 neoantigens. The great number of missense mutations results in a great number of predicted neoantigens. The same two female patients had maximum neoantigens, 2,121 and 4,896, respectively.

Patient S0616092301 only had nine missense mutations and four predicted neoantigens, while patient S0616102801 had 1,672 missense mutations and 4,896 neoantigens. Despite great difference of mutation burden between minimum and maximum, these are two nonsmoking female patients both at their fifties diagnosed with T4b stage of gastric cancer.

### 3.2. C>A and C>T Substitutions Are Major Mutation Types in Gastric Cancer

We have observed an average of 232 nonsynonymous mutations in GC. In comparison with a study from Bi* et al.*, we found that nonsynonymous mutation counts in GC were significantly higher than meningioma, thyroid cancer, pituitary adenoma, craniopharyngioma, breast cancer, and glioblastoma [21]. Our result was also in accordance with mutational signatures generated by Alexandrov* et al.* [22, 23].

**Figure 2:**
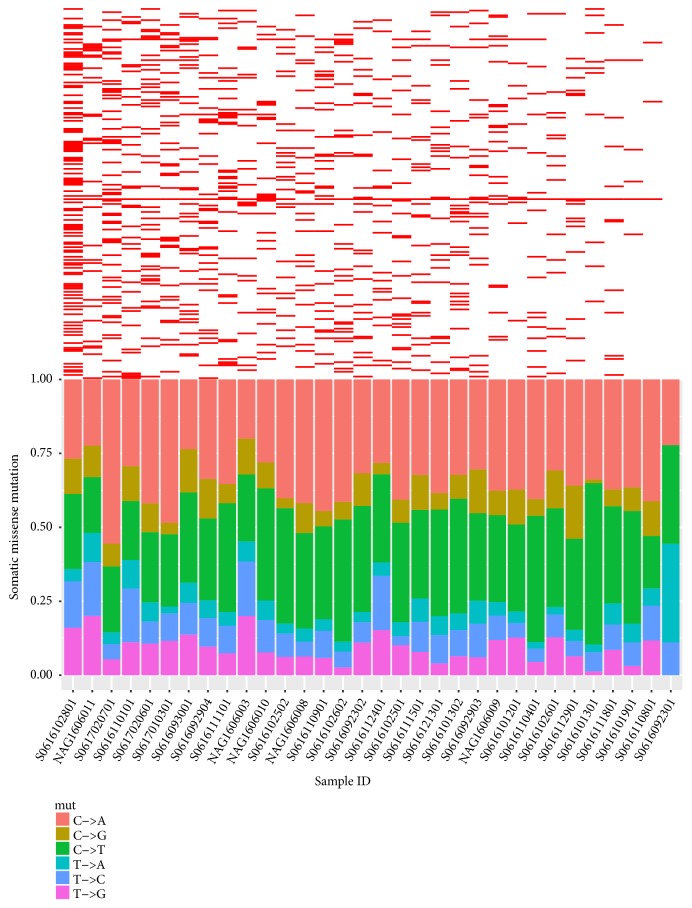
Mutational characteristics for 32 gastric cancers: upper: heat map for distribution of 232 recurrently mutated genes in 32 GC patients; bottom: percentage of missense substitution types; C>A and C>T are the major types in 32 GC patients.

We analyzed nucleotide substitution types of 7,432 missense mutations and found that 60.47% of missense mutations are transversions and 39.53% of substitutions are transitions. Individual types of substitution were presented at the bottom of Figure 2. On average, the percentage of C>A type is 32.18%, 27.24% for C>T, 12.51% for T>G, 12.29% for T>C, 9.89% for C>G, and 5.89% for T>A. C>A and C>T became the major substitution types in missense somatic mutations.

COSMIC has provided a set of 30 mutational signatures based on a large-scale analysis across nearly 40 human cancer types. 11 of 30 mutational signatures are reported related to gastric cancer. The dominant prevalence of C>A and C>T suggested a hyperactive deamination and transcribed strand bias during transcription-coupled nucleotide excision repair in gastric cancer.

### 3.3. Twelve Recurrently Mutated Genes in Gastric Cancer

The total 7,432 missense mutations were distributed in 4,451 genes. Firstly, we filtered the genes that had been discovered to be mutated at least in three patients. The number of genes decreased to 232 (see Additional file 1: Table S1). The upper part of Figure 2 presented a heat map of 232 mutant genes distributed in 32 patients. The most recurrently mutated gene was* MUC4* with an occurrence of 93.75% in this study and more than 8 nonsynonymous mutations per patient. Mucin 4 is an integral membrane glycoprotein. As major constituents of mucus, Mucin 4 plays important roles in the protection of epithelial cells in the colon, cervix, and trachea. Silencing or reduced expression of* MUC4* is associated with proliferation of pancreatic carcinoma cell line [24] and poor prognosis in renal cell carcinoma and breast carcinogenesis [25, 26]. However,* MUC4* has not been yet considered as a cancer-related gene in COSMIC.

We then matched these 232 recurrent genes to the Cancer Gene Census; only 12 genes were filtered as essential tumor-related genes:* TP53* (n=9, 28.13%),* LRP1B* (n=8, 25%),* PREX2* (n=6, 18.75%),* NRG1* (n=4, 12.5%),* PCM1* (n=3, 9.38%),* BRCA2* (n=3, 9.38%),* NOTCH1* (n=3, 9.38%),* USP6* (n=3, 9.38%),* PIK3CA* (n=3, 9.38%),* GNAQ* (n=3, 9.38%),* FAT4* (n=3, 9.38%), and* CDH1* (n=3, 9.38%).* PREX2* and* PIK3CA* are reported as famous oncogenes. By inhibition of* PTEN* activity,* PREX2* interacts with* PIK3CA* signaling pathway. In the four gastric cancer subtypes classified by TCGA,* PIK3CA* mutations occur at a frequency between 3% and 42% [12].* TP53*,* NOTCH1*,* FAT4*,* CDH1*, and* BRCA2* are tumor suppressor genes (TSG).* TP53* somatic mutations were observed in 71% of chromosomal instability (CIN) subtypes and* CDH1* mutations were enriched in 37% of genomically stable (GS) subtype [12].

### 3.4. Neoantigen Profiling of 32 Gastric Cancer Patients Revealed Significant Differences between Stages

**Figure 3:**
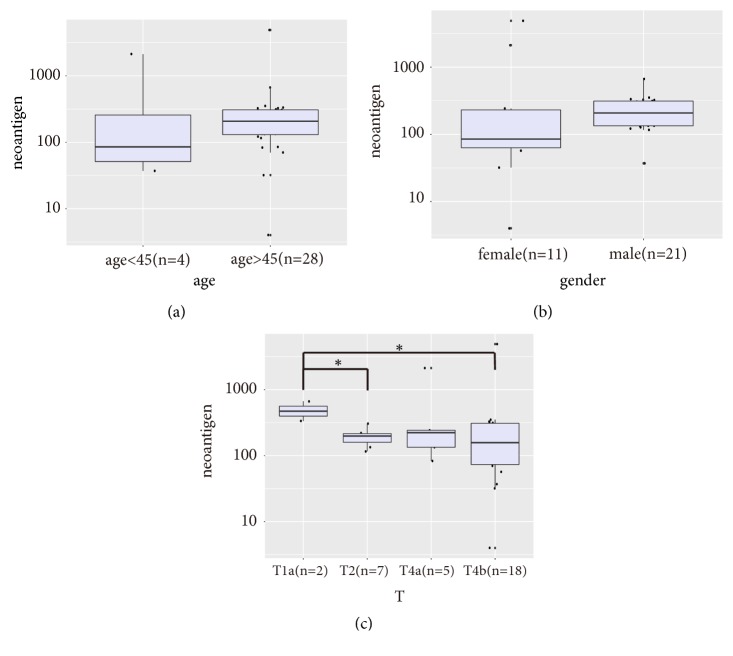
The boxplot of neoantigens' number subgrouping by age, gender, and stages. (a) The patients below 45 years (n=4) and patients above 45 years (n=28) showed no significant difference (p=0.1525). (b) The female patients (n=11) and male patients (n=21) showed no significant difference (p=0.0795). (c) The number of neoantigens differed among T1a (n=2), T2 (n=7), T4a (n=5), and T4b (n=18); the difference was significant between T1a and T2 (p=0.0202) and between T1a and T4b (p=0.0294).

We tried to study the feature of neoantigens' number associated with patients' clinical characteristics (see Figure 3). Our study enrolled 11 female and 21 male patients; the median number of neoantigens for male patients was higher than that for female patients. The patients below 45 years of age (n=4) had less predicted neoantigens than those elder than 45 years (n=28). However, the difference was not statistically significant.

GC patients are usually diagnosed at more advanced stages of cancer progression. Of the 32 patients studied, 23 were diagnosed at T4 stage. Only 2 patients were diagnosed at T1 stage and 7 patients at T2 stage. Unexpectedly, the number of neoantigens was significantly higher in T1a than in T2 (p=0.02) and T4b (p=0.03) respectively. The reason why neoantigens are less common at later stages might be due to the enrichment of dominant malignant subclones as the tumor progressed. We continued to enroll early-stage patients to see that this pattern is statistically significant.

### 3.5. Six Recurrently Mutated Genes Encoding Neoantigens Predicted to Be Potential Targets against GC

Based on the HLA genotype (see Additional file 2: Table S2) and missense mutations of 32 patients, TSNAD performed neoantigen profiling according to the affinity of mutant peptides and HLA class I molecules. The alleles HLA-A*∗*11:01 (46.9%), HLA-C*∗*01:02 (37.5%), HLA-A*∗*03:01 (25%), HLA-A*∗*24:02 (25%), and HLA-B*∗*40:01 (21.9%) were the most frequent alleles in our study. The diversity of HLA was similar to the research carried out by Gourraud* et al. *on 90 Han Chinese from Beijing dataset of the 1000 Genomes Project [27].

**Figure 4:**
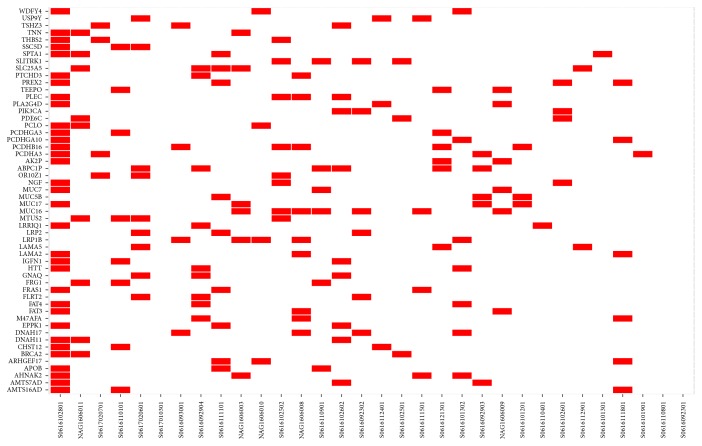
Heat map of individual distribution of 54 recurrently mutated genes which encode neoantigens.

From 12,929 predicted neoantigens, we focused on the recurrently mutated genes encoding neoantigens that were present in at least 3 patients. 54 genes were filtered, and a heat map was made to visualize the distribution (see Figure 4). Then we matched 54 genes to the cancer Gene Census.* PIK3CA*,* FAT4*,* BRCA2*,* GNAQ*,* LRP1B*, and* PREX2* were found as genes highly associated with cancer development.

The detailed mutations, HLA alleles, and sequences of 139 neoantigens derived from these six mutated genes were listed in Table S3 (see Additional file 3). The missense mutations found in* PIK3CA*,* FAT4*,* BRCA2*,* LRP1B*, and* PREX2* were not recurrent in our study and the sequences of predicted neoantigens were unique. The amino acid change of T96S in* GNAQ* was the only same mutation found in three patients and the mutant peptides were predicted to bind to HLA-A*∗*02:01, HLA-A*∗*03:01, and HLA-A*∗*11:01 alleles showing a strong binding ability (affinity IC50 <100 nM). Unfortunately, the predicted neoantigen sequences for T96S in* GNAQ* were not identical for patients because of different individual HLA-A genotype.

Due to the small size of enrolment, we used the mutation frequency published by the ICGC Project as a reference. We found that mutations S37L (1/12,198) and N289H (3/12,198) in* BRCA2*, Q453L (5/12,198) and A807V (6/12,198) in* FAT4*, T96S (10/12,198) in* GNAQ*, and H1047Y (8/12,198) and V344M (5/12,198) in* PIK3CA* have been already reported by the ICGC dataset (see Table 2) despite a very low frequency of occurrence.* PIK3CA* and its signaling pathway have been widely studied in many cancers. H1047 is a hotspot mutation in* PIK3CA*. Alternation from histidine to arginine has 281 recurrences of 12,198 donors in ICGC database. Our patient S0616102601 had the same position mutation but with an alteration from histidine to tyrosine. Only 1 donor from the ICGC gastric cancer dataset exhibited an identical mutation. In addition, we found two germline mutations in* BRCA2* (A2466V and N372H). In ClinVar, A2466V and N372H are considered as benign mutations in familial breast cancer. N372H is also annotated as a variant of unknown significance in Online Mendelian Inheritance in Man (OMIM).

The development of effective therapies against cancer has been a longstanding goal for many decades. Improvements in anticancer therapies rely on deeper understanding of the oncology and molecular basis of cancer. Recent positive developments in immunotherapy targeting neoantigens show promise as an effective method to treat cancer.

Neoantigens-associated immunotherapy could be a feasible strategy for treating malignancy with somatic mutations in driver genes encoding intracellular protein such as* KRAS*.* KRAS* has been considered as “undruggable” because of lack of binding pocket for the past 30 years. The famous amino acid alternation of Gly12 (G12V, G12C, or G12D) of* KRAS* protein is involved in 60-70% of pancreatic cancers and 20-30% of colorectal cancers [28]. Work from Rosenberg and Tran has reported impressive response from G12D-positive metastatic colorectal cancer patients when treated with autologous T-cell therapy using the HLA-C*∗*08:02 allele [29]. Of the 7 patients treated, 6 patients were reported to respond positively and are currently in remission. Mutations on* KRAS* are also an important cause for* EGFR*-targeted tyrosine kinase inhibitor (TKI) drug resistance. Moreover, the mutant peptides derived from V600E in* BRAF* [30, 31] or T790M in* EGFR* [32] were reported as binding with HLA-A*∗*02:01 and presented as neoantigens. Therefore, neoantigens-associated immunotherapy could be employed to treat cancers exhibiting common drug resistance mutations.

In the top 20 recurrently mutated genes of three ICGC projects (GACA-CN, GACA-JP, and STAD-US), seven genes were shared between Chinese and Japanese gastric cancer patients. This included* TP53*,* LRP1B*,* KMT2C*,* KMT2D*,* APC*,* GRM3*, and* SETD1B*. Meanwhile, there are eight genes (*TP53*,* LRP1B*,* KMT2C*,* KMT2D*,* APC*,* ATRX*,* NOTCH1*, and* SETBP1*) shared between Chinese and American patients. Nine genes (*NKX2*-1,* PTPN11*,* ATM*,* COLSA1*,* EPHA3*,* FOXO1*,* MSN*,* MYH11*, and* TSC1*) are unique to the Chinese patients. Our results revealed 12 cancer-related genes (*TP53*,* LRP1B*,* PREX2*,* NRG1*,* PCM1*,* BRCA2*,* NOTCH1*,* USP6*,* PIK3CA*,* GNAQ*,* FAT4*, and* CDH1*) that are recurrently mutated in GC.* TP53*,* PIK3CA*, and* CDH1* are already reported to be mutated with high prevalence in GC according to TCGA and ACRG.* FAT4* acts as a tumor suppressor by modulating Wnt/ß-catenin signaling pathway in GC [33]. Chen found that* NRG1* was mutated in 10% of 78 GC patients, and 8% of patients with mutations in* BRCA2* were associated with longer survival [34]. Aberrant methylation of* LRP1B* [35] and mutations on LDL receptor-related protein 1B cause* SMAD4*-induced GC growth [36].* PREX2* is involved in* PIK3CA*-*PTEN*-*AKT* signaling pathway. The frequency of* GNAQ* mutations was higher in intestinal-type gastric cancer [37]. Suppression of* NOTCH1* signaling pathway could induce GC progression, drug resistance, and metastasis [38, 39]. The function of* PCM1* and* USP6* in gastric cancer remains exploitable.

Recurrent oncogenic mutations such as S37L and N289H in* BRCA2*, Q453L and A807V in* FAT4*, T96S in* GNAQ*, and H1047Y and V344M in* PIK3CA* were predicted to bind with HLA-A02:01, A03:01, A11:01, B15:01, B15:02, B58:01, B40:01, B39:01, and C03:02. Unlike the distribution percentage in African or Caucasian population, A02:01, A11:01, B58:01, B40:01, B15:01, and C03:02 HLA alleles are present in more than 5% of the Han Chinese population. The possibility to discover a common neoantigen target predicted by identical HLA allele and oncogenic mutation in Chinese patients is quite higher than African or Caucasian population.

While it is unknown why some individuals fail to respond favourably from T-cell-based neoantigen immunotherapy, targeting recurrent mutations of driver genes with HLA alleles still represents a promising avenue to treat eligible patients.

In this study, we analyzed the clinical features of Chinese GC patients and paired them with the somatic mutations and neoantigens present. We chose some reoccurring neoantigens and their associated oncogenes shared by several patients for the continued development of T-cell-based immunotherapy, such as vaccines. Due to the limited sample size of our study, we included the mutation frequencies from ICGC Project in our analysis to determine recurrent oncogenic mutations. Further studies should be conducted to confirm these results.

## 4. Conclusions

In conclusion, twelve recurrently mutated driver genes were identified in our study to further understand the mechanism of GC development. To identify the “druggable” targets, neoantigen profiling by TSNAD was done, highlighting several recurrent oncogenic driver mutations. Mutant peptides encoded by seven recurrent oncogenic mutations were predicted to bind with high frequency HLA alleles as tumor-specific neoantigens. These neoantigens are currently undergoing further experimental validation as potential targets for autologous T-cell immunotherapy to treat GC patients.

## Figures and Tables

**Table 1:** The characteristics of patients and the number of mutations/neoantigens in 32 gastric cancer patients.

Sample ID	Gender	Age	TNM	T	Number of missense mutations	Number of indels	Number of neoantigens
NAG1606003	M	71	T4bN2M1	T4b	190	11	323
NAG1606008	M	80	T4bN2M0	T4b	158	35	208
NAG1606009	M	62	T4bN1M0	T4b	109	7	133
NAG1606010	M	68	T2N0M0	T2	182	20	134
NAG1606011	F	45	T4aN3bM0	T4a	1336	59	2121
S0616092301	F	54	T4bN1M0	T4b	9	5	4
S0616092302	F	58	T4aN1M0	T4a	145	31	242
S0616092903	M	61	T2N1M0	T2	115	8	190
S0616092904	M	76	T4bN3aM0	T4b	217	12	297
S0616093001	M	67	T4aN1M	T4a	233	24	223
S0616101201	M	65	T4aN0M0	T4a	102	17	134
S0616101301	M	38	T4bN2M0	T4b	77	9	37
S0616101302	M	73	T4bN1M0	T4b	124	17	351
S0616101901	F	52	T4aN2M0	T4a	63	12	83
S0616102501	F	57	T2N2M0	T2	128	16	220
S0616102502	M	66	T1aN1M0	T1a	177	9	666
S0616102601	F	62	T4bN2M0	T4b	78	9	70
S0616102602	M	49	T4bN2M0	T4b	150	14	206
S0616102801	F	50	T4bNM0	T4b	1672	104	4896
S0616110101	M	64	T4bN3aM0	T4b	331	43	313
S0616110401	F	41	T4bN3aM0	T4b	89	9	57
S0616110801	F	51	T4Bn3aM0	T4b	17	14	32
S0616110901	F	50	T2N3aM0	T2	153	6	198
S0616111101	M	59	T1aN1M0	T1a	215	8	334
S0616111501	M	62	T4bN1M0	T4b	127	7	186
S0616111801	F	60	T4bN3aM0	T4b	70	9	85
S0616112401	M	58	T2N3bM0	T2	131	7	211
S0616112901	M	54	T4bN1M0	T4b	78	22	122
S0616121301	M	42	T4bN3aM0	T4b	125	9	127
S0617010301	M	64	T2N2M0	T2	258	16	116
S0617020601	M	60	T2N1M0	T2	308	39	306
S0617020701	M	63	T4bN2M1	T4b	427	50	327

**Table 2:** The list of mutations of six recurrently mutated genes, mutation position, number of patients affected, corresponding patient HLA allele, number of predicted neoantigens, and number of patients affected in ICGC project.

Gene	Mutation in Protein Position	Number of patients affected in this study (total 32)	Patient HLA Allele	Number of predicted neoantigens	Number of patients affected in ICGC (total 12,198)
*BRCA2*	I27V	1	B58:01 C03:02 C08:01	6	0
	S37L	1	B15:02 B58:01 C03:02	6	1
	V144F	1	B15:02 C03:02	5	0
	Q147R	1	A33:03	2	0
	D156E	1	A11:01	1	0
	N289H	1	A02:01 C03:02	5	3
	T2542M	1	C14:02	2	0

*FAT4*	Q453L	1	A02:01 C03:02	12	5
	V462E	1	B40:01 B58:01 C03:02	11	0
	D598Y	1	A02:01 B15:01	5	0
	A807V	1	A02:01 B40:01 B58:01 C03:02	13	6

*GNAQ*	D95Y	1	A24:10 B18:02 B39:01 C07:02	8	0
	T96S	3	A02:01 A03:01 A11:01 B15:01 B39:01	25	10

*LRP1B*	H4368Q	1	A11:02	1	0
	L1995M	1	C03:04	1	0
	R3026S	1	A03:01 B15:01	3	0
	R4062K	1	A11:01 A33:03 C03:02	8	0
	T2206I	1	A02:01	1	0

*PIK3CA*	G106C	1	A03:01 A11:01	2	0
	H1047Y	1	B15:01	1	8
	V344M	1	A03:01 A11:01 C14:02	4	5

*PREX2*	E1428K	1	A30:01	2	0
	H895Q	1	B15:01 C03:03	3	0
	Q102H	1	B15:01	1	0
	S1488L	1	A02:01 A33:03 B40:01 C07:02	10	0

## Data Availability

The data used to support the findings of this study are available from the corresponding author upon request.

## Supplementary Materials

Supplementary 1 Additional file 1: Table S1: the distribution of 40 recurrently mutated genes in 32 GC patients.

Supplementary 2 Additional file 2: Table S2: HLA typing of 32 gastric cancer patients.

Supplementary 3 Additional file 3: Table S3: the list of predicted neoantigens in 32 gastric cancer patients.
